# Navigating AI deployment in precision livestock farming: current trends and future prospects

**DOI:** 10.1093/af/vfaf050

**Published:** 2025-12-18

**Authors:** Chuanyi Guo, Zheng He, Mutian Niu, Kai Liu

**Affiliations:** Department of Infectious Diseases and Public Health, Jockey Club College of Veterinary Medicine and Life Sciences, City University of Hong Kong, Hong Kong SAR, China; Department of Environmental Systems Science, Animal Nutrition, Institute of Agricultural Sciences, ETH Zürich, Zürich, Switzerland; Department of Infectious Diseases and Public Health, Jockey Club College of Veterinary Medicine and Life Sciences, City University of Hong Kong, Hong Kong SAR, China; Department of Environmental Systems Science, Animal Nutrition, Institute of Agricultural Sciences, ETH Zürich, Zürich, Switzerland; Department of Infectious Diseases and Public Health, Jockey Club College of Veterinary Medicine and Life Sciences, City University of Hong Kong, Hong Kong SAR, China

**Keywords:** artificial intelligence, cloud-edge computing, deployment pathways, precision livestock farming, real-time monitoring

Implications

- The selection of an AI deployment model is a critical strategic decision for livestock operations, as no single solution fits all scenarios.
- Cloud-edge collaborative architecture is emerging as the most effective paradigm, balancing on-farm responsiveness with powerful cloud analytics.
- Widespread AI adoption relies on overcoming key real-world barriers, including rural connectivity, implementation costs, and the on-farm technical skills gap.
- Future PLF advancements will depend on integrating multi-modal data to create more holistic and prescriptive animal health and welfare management systems.

## Introduction

Precision livestock farming (PLF) is undergoing a profound transformation, with its core driver shifting from traditional data collection to intelligent decision-support systems powered by artificial intelligence (AI). While early-stage PLF relied on simple sensors for discrete tasks like estrus detection, rapid advancements in the Internet of Things (IoT), sensor technology, and computing power now enable modern systems to gather vast, multidimensional data covering animal behavior, physiology, and their micro-environment (Alexy & Haidegger, 2022; Kaur et al., 2023). This evolution is driven by multiple pressures facing the global livestock industry: rising labor costs and shortages compel farms to seek automation for efficiency, while increasing consumer and regulatory demands for product quality, animal welfare, and sustainability necessitate more refined management methods. Reflecting this momentum, the global PLF market is projected to expand at a compound annual growth rate of over 10% through the next decade, signaling strong and sustained industry adoption (Sojitra & Dudhagara, 2023). Consequently, AI’s role has evolved from a frontier concept to an indispensable engine for industry advancement.

The proliferation of data has catalyzed a surge in academic research focused on developing sophisticated AI algorithms to enhance livestock production, health, and welfare (He et al., 2025). These studies have demonstrated significant potential, with models capable of predicting metabolic diseases (Giannuzzi et al., 2023), detecting specific behaviors with superhuman accuracy (Kang et al., 2020), and optimizing feeding strategies (King et al., 2024). However, the majority of this research has concentrated on algorithmic innovation and validation in controlled environments. A critical gap persists between the development of high-performing algorithms and their practical, scalable, and robust implementation on commercial farms (Berckmans, 2017). The crucial questions of how these AI systems are deployed, the architectural trade-offs involved, and the real-world challenges encountered often remain underexplored. This disconnect hinders the translation of technological potential into tangible on-farm value.

This review offers an insightful overview and future perspective on the primary AI deployment pathways in PLF, with a practical, application-driven approach. We will systematically dissect the mainstream architectures, including offline analysis, on-premises servers, edge computing, cloud platforms, and emerging cloud-edge collaborative frameworks. By examining the inherent advantages, limitations, and practical trade-offs of each pathway through recent case studies, this review will illuminate the critical challenges hindering widespread adoption, such as latency, connectivity, and data privacy. Ultimately, this article will offer a forward-looking perspective on future ­developments, providing valuable guidance for researchers, technology developers, and industry practitioners working to build the next generation of effective and accessible AI solutions for modern livestock farming.

## Mainstream AI Deployment Pathways in Precision Livestock Farming

The deployment of AI in PLF is not a monolithic practice but exists along a diverse spectrum. This spectrum ranges from fully farm-controlled, capital-intensive on-premises systems to highly flexible, service-dependent cloud solutions, with various hybrid models in between. The selection of a deployment model is therefore not merely a technical decision but a strategic one, reflecting a farm’s operational scale, capital resources, technical capabilities, and philosophy on data as a core asset.

This decision-making process is an intricate exercise in trade-offs. For instance, a small family farm with limited capital and no specialized IT staff is unlikely to build and maintain an expensive on-premises server, which demands significant upfront investment and continuous professional oversight. For such operations, low-cost, user-friendly mobile applications or pay-as-you-go cloud services represent a more realistic and accessible entry point (Shwetabhand & Ambhaikar, 2024). Conversely, a large, vertically integrated agricultural corporation may view its farm data as a key competitive advantage. Driven by concerns over data security, privacy, and ownership, and to comply with stringent internal governance or regional regulations, such an enterprise would likely invest in a private on-premises or hybrid system to ensure sensitive data never leaves the farm’s physical or virtual perimeter (Moreira et al., 2024). Geographical location and infrastructural conditions are equally decisive factors. For farms in remote areas with unstable or limited internet connectivity, a purely cloud-dependent solution is unfeasible. In these scenarios, edge computing or a cloud-edge collaborative architecture, which can perform critical data processing locally, becomes a necessity for ensuring system reliability (Batistatos et al., 2025).

**Figure 1.**
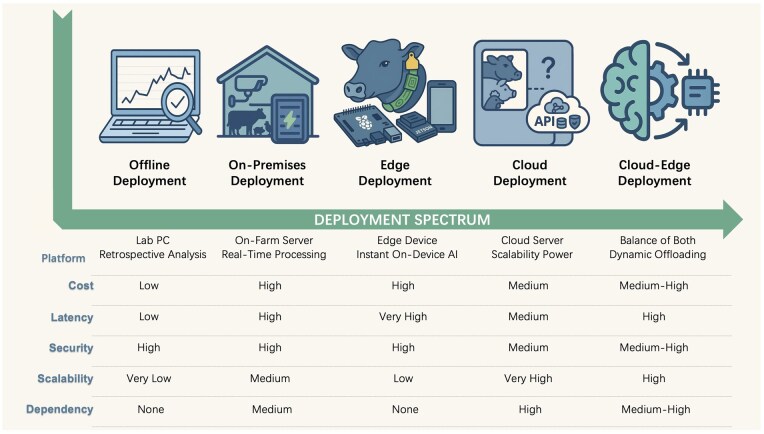
Comparative analysis of mainstream AI deployment pathways in precision livestock farming. The table evaluates the various deployment models across six key dimensions: platform, cost, latency, security, scalability, and dependency.

Consequently, a nuanced understanding of the logic and trade-offs inherent to each deployment pathway is essential. The critical consideration shifts from identifying a universally “best” technology to selecting the most suitable architecture for a specific operational context. This section provides a systematic analysis of the five mainstream deployment pathways along this spectrum, which are visually summarized in Figure 1.

**Figure 2.**
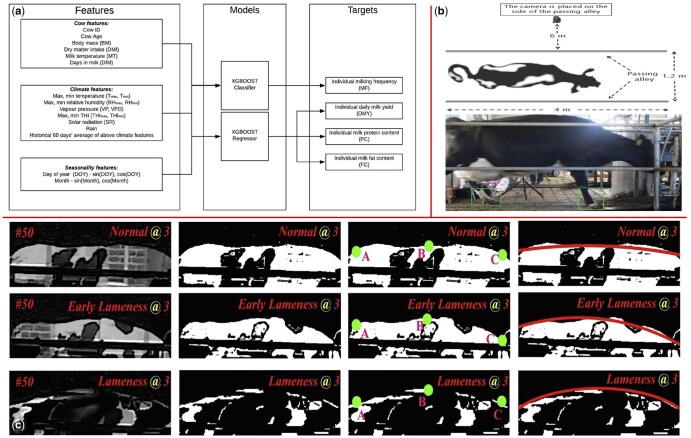
Example of offline AI analyses performed on precollected datasets for production forecasting and health monitoring. (a) Ji et al. (2022) show the prediction of future milk yield from historical records. (b) Kang et al. (2020) and (c) Jiang et al. (2022) demonstrate different computer vision approaches for post-hoc lameness detection, analyzing back curvature and hoof supporting phase from video data.

**Figure 3.**
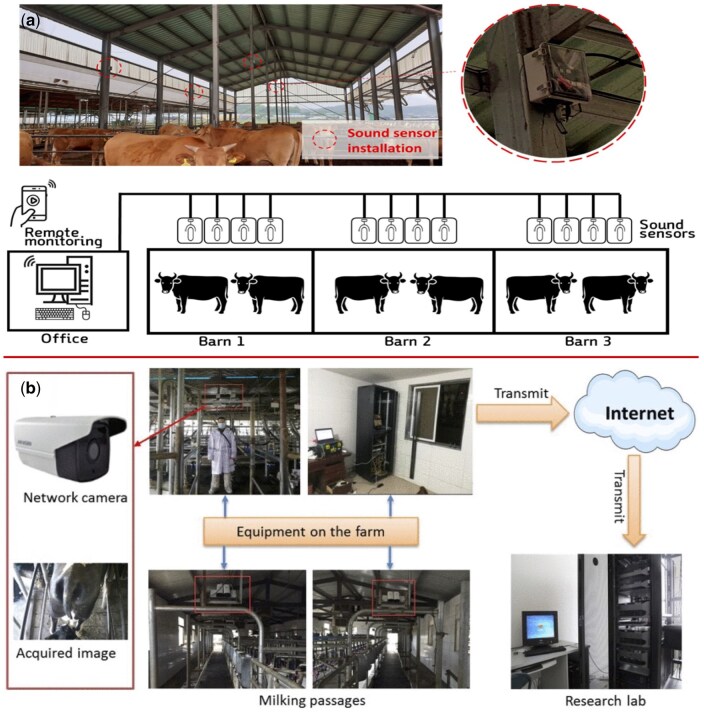
Examples of on-premises AI deployment for real-time monitoring. (a) Jung et al. (2021) illustrate a system where audio data from microphones is processed on a local PC for cattle vocalization analysis. (b) Huang et al. (2023) show a vision-based system where camera data is transmitted to an in-house server for real-time cow tail tracking.

**Figure 4.**
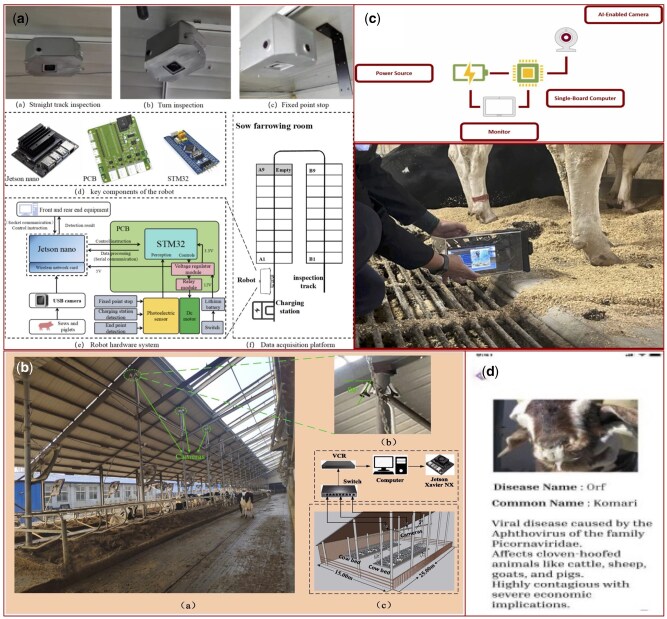
Example of studies using edge deployment for real-time, on-device animal monitoring and health diagnostics. (a) Zhou et al. (2024) show the workflow for swine behavior analysis using a Jetson Nano; (b) Xiao et al. (2024) illustrate a system for cow identification on a Jetson Xavier NX; (c) Aravamuthan et al. (2024) detail a portable device for digital dermatitis detection; and (d) Kingsley et al. (2025) presents a mobile application for goat disease detection.

**Figure 5.**
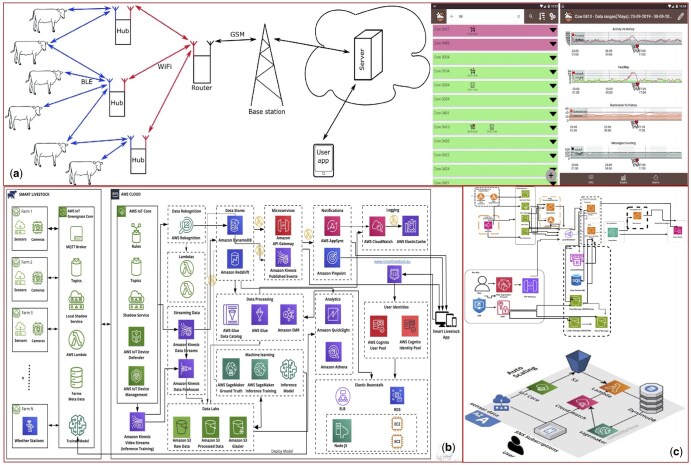
Examples of cloud-based deployment architecture for scalable livestock monitoring. (a) Unold et al. (2020) illustrate a general cloud system, while (b) Dineva and Atanasova (2021) and (c) Bhaskaran et al. (2024) showcase specific scalable architectures built on Amazon Web Services (AWS) for smart livestock management and real-time health alerts.

**Figure 6.**
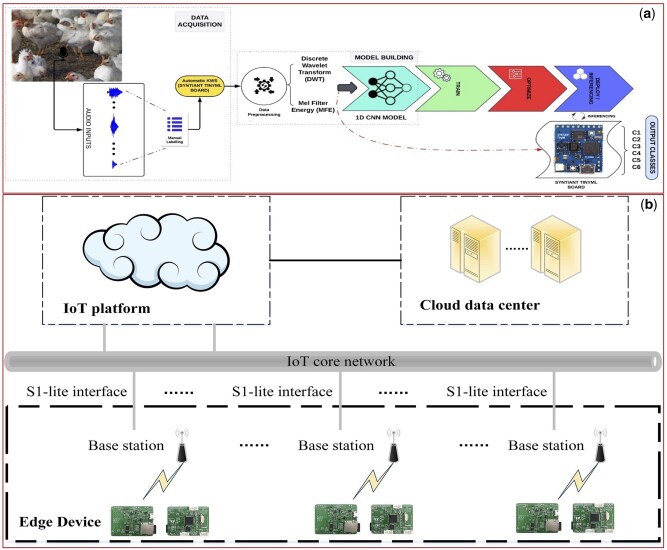
Examples of cloud-edge collaborative deployment architecture. (a) Srinivasagan et al. (2025) illustrate a workflow where a model is trained in the cloud and deployed on a low-power edge device for real-time inference. (b) Shen et al. (2021) show a system where the edge device performs local data processing and classification, sending only the results to the cloud for long-term aggregation.

### Offline deployment: post hoc analysis of historical data

Offline deployment represents a foundational and widely adopted paradigm for applying AI in PLF, characterized by its “collect-first, analyze-later” approach (Figure 2). In this pathway, farms systematically accumulate data over extended periods, forming comprehensive historical datasets that are subsequently used to train and validate machine learning models in a nonreal-time environment. This decoupling of model development from daily farm operations allows for deep, retrospective analysis aimed at informing long-term strategic decisions rather than immediate interventions.

This deployment model is prevalent in academic research and has been successfully applied to address key challenges using various data types. For tabular and sensor data, offline models have demonstrated significant predictive power. For example, Perneel et al. (2024) successfully explained up to 47% of the variance (*R*^2^) in a cow’s lifetime production potential by analyzing historical genetic and environmental records using stacking ensemble models. Similarly, a random forest model developed using 20 years of test-day records was able to forecast early-lactation milk yield with a root mean square error between 6.08 and 6.24 kg (Salamone et al., 2022). In health applications, high accuracy has been achieved in predicting blood metabolites from milk infrared spectra (Giannuzzi et al., 2023), while other models have effectively predicted insemination outcomes (Shahinfar et al., 2014) and forecasted future milk yield (Ji et al., 2022).

Vision-based analysis is another prominent domain for offline deployment, where extensive video or image data is processed post-hoc (Oliveira et al., 2021). In lameness detection, for instance, Jiang et al. (2022) developed sophisticated deep learning pipelines that combine custom object detection with network models to classify lameness from back curvature data with 96.61% accuracy. Another approach analyzed the hoof supporting phase using a deep learning network, resulting in 96% classification accuracy (Kang et al., 2020). This method has also proven effective for monitoring feeding behavior, as a study by Bresolin et al. (2023) trained a YOLOv3 deep learning model on annotated historical images to achieve 96.0% accuracy in individual heifer identification, which in turn enabled the precise calculation of feeding time (*R*^2^ = 0.99).

A primary advantage of offline deployment is its minimal requirement for on-farm real-time infrastructure, which lowers the barrier for adoption. It allows for the use of large-scale, longitudinal datasets and computationally intensive algorithms to build robust models that support strategic planning. However, the principal limitation of this pathway is its inherent lack of real-time actionability. Models cannot provide immediate alerts for acute health events, and insights are generated retrospectively, meaning the optimal window for intervention may have already passed. Consequently, models trained exclusively on historical data may become less accurate as farm conditions evolve, positioning this approach as a reactive, analytical tool rather than a proactive, real-time management system.

### On-premises deployment: real-time processing on in-house servers

On-premises deployment marks a significant step from offline analysis toward real-time farm management (Figure 3). This pathway involves installing local computing hardware, such as servers or dedicated PCs, within the farm’s infrastructure. Data from cameras or microphones is transmitted over a local network to these in-house servers for immediate processing. The core advantage of this model is its ability to run AI algorithms in real-time or near-real-time, generating immediate alerts and management insights without relying on an external internet connection for the primary computational tasks. This enables proactive monitoring and timely interventions.

The application of on-premises systems is particularly valuable for monitoring dynamic events where immediate feedback is critical. A clear example of this architecture can be seen in audio-based monitoring, where a system used microphones in a barn to capture cattle vocalizations. The sounds were sent to a central on-premises PC that ran a convolutional neural network (CNN) in real-time, achieving up to 94.18% accuracy in distinguishing cattle calls from background noise and providing instant alerts for events like estrus or distress (Jung et al., 2021).

In vision-based behavior and health monitoring, the feasibility of on-premises deployment hinges on the computational efficiency of the AI models. Several studies have developed lightweight yet powerful algorithms suitable for this purpose. For instance, high accuracies (97.87% for eating and 98.27% for drinking) have been achieved in recognizing goat behaviors using a YOLOv4-based system that processes 17 frames per second (FPS) on conventional hardware, well within the capacity of a local server (Jiang et al., 2020). More advanced tracking systems for pigs have reached speeds of over 70 FPS (Tu et al., 2024), and an improved Single Shot Multibox Detector model for cow tail tracking achieved 96 FPS. Although tested in a lab, the high speed of this latter model demonstrates its readiness for on-premises deployment, where it could provide real-time indicators for calving or health status (Huang et al., 2023).

The primary strength of on-premises deployment is its capacity for immediate feedback, enabling farmers to intervene quickly. This model also offers superior data privacy and security, as all information is processed and stored locally. Furthermore, it ensures operational reliability, functioning independently of potentially unstable rural internet connections. However, this approach comes with significant drawbacks. The initial capital expenditure for server hardware can be substantial, and these systems require ongoing on-site technical maintenance. Scalability also presents a challenge, as expanding the system often necessitates costly hardware upgrades, in contrast to the more flexible and cost-effective scaling offered by cloud-based solutions.

### Edge deployment: instant intelligence in smart devices

Edge deployment represents the most decentralized and responsive pathway for AI in PLF, where intelligence is moved from central servers directly onto the data-capturing devices themselves (Figure 4). In this model, AI algorithms run on resource-constrained hardware, such as embedded systems (e.g., NVIDIA Jetson, Raspberry Pi) integrated into smart cameras or mobile devices. This allows for immediate, on-device processing of data at the source, eliminating the need for continuous data transmission to a local server or the cloud and enabling instantaneous feedback.

The viability of edge deployment hinges on the development of highly efficient, lightweight AI models that can perform complex tasks with minimal computational resources. Recent advancements have made this increasingly feasible for real-time monitoring. In swine behavior analysis, for example, Zhou et al. (2024) successfully deployed a YOLOv8n model for sow posture detection and a TSM_MobileNetv2 model for piglet activity recognition onto a Jetson Nano. After optimization with TensorRT, the YOLOv8n model achieved an impressive processing speed of 36.4 FPS on the edge device, while the video analysis model processed clips in just 542.51 ms. Similarly, real-time individual cow identification at 20 FPS has been achieved by deploying a YOLOX-s and a custom CowbodyNet model on a Jetson Xavier NX (Xiao et al., 2024).

Edge deployment is also proving effective for real-time health diagnostics. A portable system developed by Aravamuthan et al. (2024) for detecting digital dermatitis runs a Tiny YOLOv4 model on a Jetson Xavier NX, achieving a high-speed inference rate of 40 FPS with a Cohen’s kappa agreement of 0.830, demonstrating its clinical utility. The accessibility of edge AI extends to even lower-cost hardware and ubiquitous mobile platforms. A custom lightweight CNN designed for cattle face recognition, for instance, ran at 300 ms per image on a Raspberry Pi 4B (Li et al., 2022). Furthermore, researchers have successfully deployed applications on standard smartphones by converting models into optimized formats like TensorFlow Lite. Examples include a YOLOv5 model for digital dermatitis detection on iOS and Android, achieving inference times as low as 20 ms on an iPhone (Dwivedi et al., 2025), and a similar system using YOLOv8 for automated goat disease detection (Kingsley et al., 2025).

The primary advantage of edge deployment is its ultra-low latency, which facilitates immediate alerts and real-time control. By processing data locally, this approach drastically reduces network bandwidth requirements, leading to low operational costs. It also enhances data privacy and security as sensitive information does not need to leave the farm, and ensures high operational reliability even in areas with poor or no internet connectivity. However, this pathway also presents significant challenges. The computational constraints of edge hardware are a primary concern, as AI models must be meticulously optimized and compressed, often involving a trade-off between inference speed and predictive accuracy. This process necessitates specialized expertise. Furthermore, scalability can be an issue. Although adding new devices is straightforward, managing a large, distributed fleet introduces significant complexity and maintenance overhead compared to centralized architectures.

### Cloud deployment: scalable computing and big data analytics platforms

Cloud deployment represents a paradigm where data collected from on-farm sensors, cameras, and other IoT devices are transmitted via the internet to remote server clusters for storage, management, processing, and analysis (Figure 5). Unlike on-premises solutions that rely on local farm servers, this model leverages the virtually limitless computational power, storage capacity, and advanced analytical tools offered by cloud service providers like Amazon Web Services (AWS), Microsoft Azure, Alibaba Cloud, and Huawei Cloud. By shifting the core tasks of data processing and AI model computation from the farm to the cloud, this approach facilitates superior scalability, robust data handling, and the application of complex algorithms to derive deep insights for farm management.

The evolution of cloud technology has positioned it as a preferred solution for managing the massive and heterogeneous datasets inherent in PLF. Prime examples include comprehensive livestock health monitoring systems built on AWS (Dineva & Atanasova, 2021). In these systems, wearable sensors track key indicators such as movement, temperature, and heart rate, streaming the data to the AWS cloud. A suite of services, including AWS IoT Core for device management and Amazon S3 for storage, work in concert while machine learning models on Amazon SageMaker analyze the data to predict health issues. For instance, when an abnormal metric is detected, such as a heart rate exceeding a preset threshold of 150 bpm, the system can instantly send an alert to a farmer’s mobile device (Bhaskaran et al., 2024).

Another pioneering application by Ferreira et al. (2024) utilizes the Microsoft Azure cloud platform for the early detection of subclinical ketosis in dairy cows through multimodal data fusion. This framework successfully integrates three disparate data types: 3D depth-camera images for automated body condition scoring, wearable sensor data, and genomic single-nucleotide polymorphism markers. The entire workflow is automated in the cloud, with the image processing pipeline alone achieving a Dice similarity coefficient of 0.99 for body segmentation, 99.1% accuracy for image quality assessment, and 93.2% for individual animal identification. By fusing deep features from these varied sources, the final predictive model achieved an F1-score as high as 0.750, showcasing the cloud’s power in integrating heterogeneous data for complex disease prediction.

The primary advantage of cloud deployment is its exceptional scalability, allowing farms to expand from a few dozen to thousands of sensors without upfront hardware investment (Bhaskaran et al., 2024). It provides the immense computational power necessary for training sophisticated deep learning models and serves as a central hub for breaking down data silos, enabling remote access to dashboards and alerts (Unold et al., 2020). However, this model is fundamentally dependent on stable, high-speed internet, which can be a significant barrier in rural areas. Data transmission costs, latency for real-time control applications, and persistent concerns over data privacy on third-party servers remain key challenges to its widespread adoption.

### Cloud-edge collaborative deployment: the rise of hybrid architectures

Cloud-edge collaborative deployment represents a sophisticated hybrid architecture that strategically allocates computational tasks between on-farm edge devices and centralized cloud servers (Figure 6). This model has emerged as a leading solution to the challenges of latency, network limitations, and data volume in PLF. Unlike a pure cloud model where edge devices act merely as data collectors, in this framework, the edge performs initial, lightweight processing. The implementation of this synergy is diverse, primarily distinguished by how tasks are partitioned between the edge and the cloud.

One fundamental approach involves performing data preprocessing and feature extraction at the edge to minimize data transmission. In a system for monitoring cow rumination, for example, wearable edge devices locally process raw accelerometer data, running a lightweight algorithm to classify behavior in real-time. Only the final classification result, a simple binary output, is uploaded to the cloud for long-term aggregation. This method reduces the required data transmission volume by an astounding 99.9% while achieving a local classification accuracy of 96.1% (Shen et al., 2021).

A more advanced pathway leverages the cloud for intensive model training and optimization, while deploying the resulting lightweight models for real-time inference at the edge. This strategy is exemplified in studies on poultry vocalization and piglet behavior. For poultry welfare analysis, a 1D CNN model is trained and compressed on a cloud platform before being deployed on a low-power TinyML device in the barn, achieving an inference latency as low as 47 ms with 96.6% accuracy (Srinivasagan et al., 2025). Similarly, for recognizing piglet behaviors, a complex “teacher model” is trained in the cloud, and its intelligence is then transferred to a streamlined “student model” through knowledge distillation. When deployed on an NVIDIA Jetson Orin NX edge platform, this optimized model reduced average inference time by 53.8% to 163.2 ms (Luo et al., 2025).

The most dynamic form of collaboration involves intelligent, real-time task offloading based on computational demand. In a cattle biometric identification framework named “Dairy DigiD,” an optimized YOLOv11 model runs on an NVIDIA Jetson NX edge device. If the model’s prediction confidence is high (e.g., >85%), the classification is finalized locally. However, if the confidence is low, indicating a challenging case, the task is automatically offloaded to a more powerful DenseNet121 model in the cloud for secondary, high-precision analysis. This dynamic workflow ensures routine tasks are handled efficiently at the edge, achieving 24 FPS, while complex decisions benefit from the cloud’s superior computational power, maintaining an overall system accuracy of 94.2% (Mahato & Neethirajan, 2025).

While this hybrid model offers significant advantages, including low latency, reduced network dependency, and enhanced data privacy, its practical implementation presents considerable engineering challenges. The complexity of managing a distributed system is a primary concern, requiring robust protocols for device monitoring and software updates. Furthermore, designing effective synchronization mechanisms is critical for maintaining data consistency and resolving conflicts caused by intermittent connectivity. The challenge of model migration is also substantial. This process requires advanced technical pathways to adapt large cloud-trained models for edge hardware, often involving model format conversion (e.g., to TensorFlow Lite or ONNX) and optimization techniques such as quantization (reducing numerical precision), pruning (removing redundant model parameters), and knowledge distillation (transferring knowledge to a smaller model). These hurdles demand a high level of technical expertise and represent major obstacles to the seamless deployment of cloud-edge systems in real-world farm environments (Luo et al., 2025).

## Key Challenges and Limitations in Real Applications

The transition of AI technologies from controlled research settings to the dynamic environment of commercial farms is fraught with significant practical challenges. These barriers represent the gap between the potential of the deployment models discussed and their real-world implementation, and they must be systematically addressed to facilitate widespread adoption.

### Infrastructural and technical barriers

At the most fundamental level, the practical deployment of AI is constrained by the infrastructural and environmental realities of the farm. The efficacy of pure-cloud (Section 2.4) and cloud-edge collaborative (Section 2.5) architectures is fundamentally dependent on stable, high-bandwidth internet connectivity—a resource that remains scarce in many rural agricultural regions. This digital divide limits deployment options and complicates essential tasks like model updates (Kaur et al., 2023). Furthermore, the harsh physical conditions of farms, characterized by dust, moisture, and temperature extremes, demand robust and specially housed hardware for on-premises servers (Section 2.2) and edge devices (Section 2.3), adding to both cost and maintenance complexity.

Beyond the physical infrastructure, a significant technical skills gap presents another major operational hurdle. The deployment and maintenance of distributed AI systems, particularly sophisticated cloud-edge frameworks, require specialized expertise in IT and machine learning operations. This stands in stark contrast to the general skill set available on most farms, creating a reliance on continuous external support. The need for intuitive, automated management tools to handle tasks like model calibration and updates is therefore critical for bridging this gap.

### Economic and data governance barriers

Even if technical and infrastructural challenges are met, economic viability and a clear return on investment (ROI) remain a central concern for producers. While the technical performance of AI models can be impressive, there is a notable lack of comprehensive studies translating these metrics into tangible economic benefits, such as quantifiable reductions in mortality or feed costs. The substantial initial capital expenditure for hardware, like NVIDIA Jetson series devices, coupled with recurring operational costs for cloud services, necessitates a compelling business case that is not yet fully established with empirical financial data (Boyer et al., 2024).

Underpinning these practical considerations are core concerns of data privacy, security, and ownership. Farm operational data constitutes a valuable and sensitive commercial asset. Consequently, cloud-based deployments can trigger apprehension regarding data sovereignty and the potential for misuse by third-party providers. While on-premises and edge deployments offer greater data control, the transparency and robustness of security protocols across the entire data pipeline are crucial for building the trust necessary for adoption.

### Ethical and adoption barriers

Beyond data management, the deployment of AI in PLF introduces broader ethical dilemmas that are closely tied to the chosen architecture. For example, cloud-based models that aggregate data from numerous farms could inadvertently perpetuate algorithmic bias if training datasets are not sufficiently diverse, potentially leading to suboptimal or unfair recommendations for underrepresented farm types. Another ethical concern is the risk of increased surveillance (Bao & Xie, 2022). Although intended to improve welfare, AI systems could potentially be used to push animals to their physiological limits for productivity gains. Furthermore, the high upfront cost associated with sophisticated on-premises or edge systems could exacerbate economic disparities, creating a technological divide between large corporations and smaller family farms. These ethical dimensions require careful consideration in the design and deployment of any AI solution in the livestock sector.

Ultimately, the success of any deployed system hinges on user acceptance and human-AI trust. A sophisticated AI system is of little value if its outputs are not trusted or acted upon by farm personnel. The system’s reliability, ease of use, and the intuitive presentation of its insights are paramount. As demonstrated by Mahato and Neethirajan (2025), a well-designed user interface that reduces technician training time by 84% can be instrumental in overcoming this human-computer interaction barrier. Addressing these multifaceted challenges is crucial for moving AI in PLF from promising research to widespread, impactful reality.

## Outlook

Building on the current state of AI deployment, the future of PLF is poised for an evolution from isolated applications toward an integrated, intelligent ecosystem. This progression will be defined by several key trends that address today’s challenges and unlock new capabilities.

The most significant shift will be a move beyond the limitations of single sensor monitoring toward the integration of diverse data streams. Drawing inspiration from frameworks like that of Ferreira et al. (2024), which fused visual, sensor, and genomic data, the next generation of AI will construct a holistic digital profile for each animal. By correlating these multimodal inputs, such as camera, microphone, and wearable data, these systems can move beyond simple event detection to provide deep, contextual insights into an animal’s health, welfare, and physiological state.

Concurrently, the role of AI will advance from predictive alerts to prescriptive decision support. This marks an evolution from systems that only identify potential problems to those that recommend specific, data-driven solutions. Rather than simply issuing a generic alert for a heightened risk of lameness, future systems will provide actionable management recommendations, such as suggesting a hoof inspection based on a quantifiable decline in an animal’s gait score. This transforms AI from a passive monitoring tool into an intelligent co-pilot for veterinarians and farm managers.

The democratization of this technology will also accelerate, driven by two primary forces. First, the declining cost of powerful and energy-efficient edge processors, such as the TinyML chips used by Srinivasagan et al. (2025), will make sophisticated on-farm AI more accessible. Second, the emergence of privacy-preserving technologies like Federated Learning will be transformative. This approach allows AI models to be trained collaboratively across multiple farms without requiring the sharing of sensitive raw data, directly addressing the core data privacy dilemma and fostering greater industry-wide innovation (Mao et al., 2023).

However, technology alone cannot bridge the adoption gap. A strategic, staged adoption roadmap is crucial for producers of all scales to mitigate financial risk. For small and medium-sized farms, this may involve starting with low-cost solutions like offline analysis of existing records or deploying a single edge device to address a critical pain point and validate ROI. Large agricultural corporations can adopt a similar pilot-based strategy, implementing and comparing different deployment architectures in specific units before committing to a farm-wide, capital-intensive rollout (Wolfert et al., 2017). This phased approach ensures that technology adoption is driven by proven value, but its ultimate success also depends on a supportive external ecosystem. Public–private partnerships will be vital for de-risking innovation, modernized agricultural extension services must translate data into actionable advice, and targeted AI literacy training is essential to empower farm operators.

Finally, the landscape will mature from siloed systems to open, scalable ecosystems. The current model of proprietary, closed-off systems will give way to open and standardized platforms that promote interoperability. This shift is already being catalyzed by the proliferation of powerful open-source tools like DeepLabCut (Mathis et al., 2018) and SLEAP (Pereira et al., 2022), which provide sophisticated, markerless pose-estimation capabilities. Such a transition will allow farmers to integrate best-in-class technologies from various vendors, breaking down data silos, avoiding vendor lock-in, and fostering a more competitive and innovative market that can scale from small pilots to large, enterprise-level operations.

## Conclusion

This article has charted the trajectory of AI in PLF, confirming its transformation from a novel concept into a core engine driving the industry’s modernization. The systematic analysis of the five primary deployment pathways reveals a clear and compelling trend: while each model offers distinct advantages, the cloud-edge collaborative architecture has emerged as the most promising and practical paradigm. Its ability to synergistically balance real-time responsiveness at the edge with the immense analytical power of the cloud makes it uniquely suited to meet the complex demands of modern livestock operations.

However, the path to widespread adoption is contingent on overcoming significant hurdles. Challenges related to rural infrastructure, technical complexity, a clear ROI, and critical concerns over data security must be proactively addressed by researchers, developers, and industry stakeholders together. These are not minor obstacles but fundamental barriers that will shape the future pace of innovation.

Looking forward, the potential for AI in this domain is vast. The convergence of multimodal data fusion, prescriptive decision support, and privacy-preserving technologies will empower a new generation of intelligent farming. This future promises not only gains in production efficiency but also profound improvements in animal welfare and environmental sustainability. By continuing to bridge the gap between algorithmic potential and on-farm reality, AI is set to redefine the future of livestock agriculture, fostering a system that is more productive, humane, and resilient.

## Author Biographies

**Chuanyi Guo** is a Ph.D. candidate in the Department of Infectious Diseases and Public Health at the Jockey Club College of Veterinary Medicine and Life Sciences, City University of Hong Kong. He received his bachelor’s and master’s degrees in computer science and technology from Harbin Institute of Technology (HIT) and the Chinese University of Hong Kong (CUHK) in 2022 and 2023, respectively. His current research focuses on the application of computer vision and sensor-based machine learning for animal behavior and welfare, as well as the development of cloud-edge collaborative systems.

**Zheng He** is a Ph.D. candidate in the Department of Infectious Diseases and Public Health at the Jockey Club College of Veterinary Medicine and Life Sciences, City University of Hong Kong. He received his bachelor’s and master’s degrees in pattern recognition and intelligent system from Harbin Engineering University and Sichuan University in 2019 and 2022, respectively. His current research focuses on computer vision, deep learning, precision livestock farming, artificial intelligence for animal behavior, and welfare.

**Dr Mutian Niu** is an Assistant Professor of Animal Nutrition in the Institute of Agricultural Sciences of the Department of Environmental Systems Science, at ETH Zurich, Switzerland. His research focuses on nutritional strategies to enhance nutrient utilization efficiency, reduce environmental impact, and improve animal welfare, contributing to the long-term sustainability of dairy farming systems. With expertise in ruminant nutrition, statistics, and data science, Dr Niu’s work contributes to translational research that links fundamental discoveries with real-world applications. His current research program explores dietary strategies for enteric methane mitigation and pioneers noninvasive exhalomics approach to deepen our understanding of rumen function and physiological responses in dairy cows.

Corresponding author: mutian.niu@usys.ethz.ch

**Dr Kai Liu** is an Associate Professor specializing in precision livestock farming and animal behavior and welfare at the Jockey Club College of Veterinary Medicine and Life Sciences, City University of Hong Kong. He earned his PhD in Agricultural and Biological Engineering from Iowa State University and a bachelor’s degree in Biosystems Engineering from Zhejiang University. In 2018, he embarked on a postdoctoral research position at the Swine Teaching and Research Center at the University of Pennsylvania. As an agricultural and biological engineer deeply committed to animal agriculture, he is dedicated to applying innovative engineering strategies and data-driven solutions to address global challenges in animal production and sustainability, with a keen emphasis on the ever-growing issues related to animal welfare and behavior.

Corresponding author: kailiu@cityu.edu.hk
